# Highlights on Genetic Polymorphism Associated with Thromboembolic Risk; Implications in Ophthalmic and Autoimmune Disorders—A Review

**DOI:** 10.3390/diagnostics13071267

**Published:** 2023-03-27

**Authors:** Mihaela Oana Romanitan, Matei Popa-Cherecheanu, Victor Andrei Vasile, Simona Stanca, George Iancu, Mihail Zemba, Daniel Branisteanu, Raluca Iancu, Ruxandra Angela Pirvulescu

**Affiliations:** 1 Department for Emergency Internal Medicine and Neurology, Stockholm South General Hospital, 11883 Stockholm, Sweden; 2 Department of Ophtalmology, Faculty of Medicine, Carol Davila University of Medicine and Pharmacy, 050474 Bucharest, Romaniaruxandrapascu78@gmail.com (R.A.P.); 3 Cardiovascular Surgery Clinic, “Prof. Dr. Agrippa Ionescu” Emergency Clinical Hospital, 011356 Bucharest, Romania; 4 Department of Ophthalmology, University Emergency Hospital, 050098 Bucharest, Romania; 5 Department of Ophthalmology, Adolphe de Rothschild Foundation Hospital, 75019 Paris, France; 6 Grigore Alexandrescu Pediatric Clinical Hospital, 011743 Bucharest, Romania; 7 Filantropia Clinical Hospital, 011132 Bucharest, Romania; 8 Department of Ophthalmology, Emergency Central Military Hospital, 010825 Bucharest, Romania; 9 Department of Ophthalmology, University of Medicine and Pharmacy “Grigore T. Popa”, 700115 Iasi, Romania

**Keywords:** genetic polymorphism, thromboembolism, central retinal vein occlusion, rheumatoid polyarthritis, acute angle closure glaucoma

## Abstract

The present paper explores genetic polymorphism and its association with thromboembolic retinal venous disorders, such as central/hemi-retinal vein occlusion, as well as possible correlations with other ocular findings, such as closed angle glaucoma, but also with autoimmune general disorders. In this review, we are highlighting the importance of establishing a correspondence between all of the above, since they all have complex etiopathogeneses; sometimes, when all coexist together, they could generate effects that may be very difficult to manage. There are studies supporting that genetic polymorphism, such as the variant MTHFR A1298C, may increase the risk for developing glaucoma, especially in the heterozygote model. Being aware of all these aspects may prove to be useful in patients with several associated diseases, as a combined effort between several medical specialties may prove to the benefit of these patients. Our review, completed with an exemplifying clinical case, shows that it is necessary to raise awareness of all aspects of a complex medical situation, including the genetic one, of a patient being at risk for thromboembolic episodes, for preventing them or managing them promptly and properly in the future.

## 1. Introduction

Retinal venous occlusive disease is the second most frequent retinal vascular disorder after diabetic retinopathy, with a prevalence of 0.7%–1.6%. Depending on the site of the venous blockage, retinal vein occlusions (RVO) are classified as central retinal vein occlusion (CRVO), branch retinal vein occlusion (BRVO) and hemi-retinal vein occlusion (HRVO) [1].

The pathogenesis of RVO remains partially unclear. The condition may occur due to three combined systemic changes known as Virchow’s triad: 1. hemodynamic changes (venous stasis), 2. degenerative changes of the vessel wall, and 3. blood hypercoagulability [2].

Among the elements of Virchow’s triad, the contribution of thrombogenesis or hypercoagulability of the blood to the pathogenesis of CRVO has not been as well investigated as the other two factors. Therefore, the implications of various hematological abnormalities in the etiopathogenesis and treatment of CRVO remain partially unclear and there are contrasting clinical results [3].

## 2. Genetic Features on Retinal Vein Occlusion

Although Virchow’s triad explains the systemic changes leading to CRVO, this condition may be caused by both local and systemic causes. The etiopathogenetic mechanisms can be divided into conditions that produce a physical blockage at the level of the lamina cribrosa and conditions in which hemodynamic factors contribute to obstructing the blood flow [3,4].

When a retinal venous occlusion occurs, a thrombus develops, usually at the lamina cribrosa level (particularly when we refer to the central retinal vein), leading to the obstruction of the retinal blood supply, causing local ischemia, retinal edema and decreased visual acuity. The condition has several triggers; with age, lamina cribrosa loses its elasticity, causing a compression of the vascular wall; furthermore, degenerative processes that arise from the central retinal artery (atherosclerosis) may induce rigidity of the central retinal artery, which causes compression of the venous wall; the resulting turbulent flow disturbs blood hemodynamics and determines endoluminal thrombus formation [3,4].

In most cases, these causative mechanisms do not occur individually, they most likely coexist in many patients that develop retinal vein occlusion.

Several studies indicate that primary thrombosis is quite rare; more often, thrombosis may occur as an end-stage phenomenon, complicating other causing mechanisms in the obstructive process [5].

Retinal vein occlusion might also coexist with other risk factors, such as atherosclerosis, age, diabetes mellitus, high blood pressure, vasculitis (e.g., sarcoidosis), coagulopathies, blood hyper viscosity due to changes in the coagulating factors in the blood (genetic polymorphism, with deficiency of thrombolytic factors and/or increase in clotting factors), migraine and smoking. Most of these conditions may lead to other retinal or optic nerve disorders, making the differential diagnosis of retinal vein occlusions sometimes challenging (Table 1) [4,5].

Some studies reveal that there are about 16.4 million adults worldwide affected by any form of RVO. In most cases, thrombophilia gene mutations most correlated with RVO are the factor V (FV) Leiden gene mutation (G1691A), the factor II (FII) gene mutation (G20210A), and the 5,1-methylenetetra-hydrofolate reductase (MTHFR) gene mutation (C677T); however, there may still exist other mutations involved in RVO pathogeny. In some cases, such mutations may coexist along with some other general comorbidities, contributing to the increased risk of venous occlusion. The questions and conflicts regarding venous occlusion origins still remain unsolved despite all the studies and their results available at this moment [6].

Retinal vein occlusion (RVO) is a retinal vascular disease more common in the elderly and, frequently, may cause visual morbidity and blindness. Most cases with RVO occur in individuals over 50 years old; among those, more than 50% associate other cardiovascular comorbidities.

The clinical aspects of RVO are depending on the type and the site of occlusion (if it involves the central retinal vein, or only a venous branch). The patient may have minor complaints regarding visual acuity, or the vison may be significantly reduced. Patients may have a relatively normal exam anterior pole, or they may present an afferent pupillary defect in the affected eye and color vision may be reduced. Eye fundus examination usually reveals tortuous veins (especially the one involved), flame shaped hemorrhages, cotton wool spots and swelling of the optic disc. These signs are extensive if there is a central retinal vein occlusion (CRVO). In this case, the aspect of the ocular fundus is described as “blood and thunder” appearance, due to the extensive hemorrhages seen throughout the retina. In non-ischemic central retinal vein occlusion, an ophthalmoscopy exam typically shows tortuosity and mild dilation of the retinal veins, as well as hemorrhages in all quadrants. Ischemic CRVO typically show marked retinal edema, venous dilation and tortuosity, and extensive 4-quadrant hemorrhage [2,4].

As far as hemostatic mechanisms are concerned, there are several congenital or acquired deficiencies involved. Among the congenital deficiencies, the most important are antiphospholipid antibodies, protein C or S deficiencies and antithrombin (AT) deficiency. There are also some mutations and/or polymorphisms in genes that modulate hemostatic balance—such as FV G4070A, FV G1691A or FIIG 20210A, fibrinolysis (PAI-1-675 4G/5G), endothelial integrity and function, (EPCR A4600G and G4678C), clot stability (FXIII exon 2G/T), that are involved in hypercoagulable states and thus, may contribute to RVO onset. Another risk factor to consider is hyperhomocysteinemia; polymorphisms in genes encoding the enzymes involved in the metabolism of homocysteine (MTHFR C677T and A1298C) as well as reduced enzyme activity might also increase the risk for RVO [5,6].

Giannaki et al. (2013) conducted a study on a cohort of patients, to find if there is any possible additional effect of genetic susceptibility to the already well-known common risk factors of RVO. All 51 patients in the study were diagnosed with RVO (either CRVO or BRVO) and all of them were submitted to a detailed anamnestic investigation for hypertension, diabetes, dyslipidemia, malignancy, and any chronic medication such as antiplatelet, anticoagulants, hormones (e.g., estrogen formulations). Patients were also questioned about previous individual/family history of venous/arterial thrombotic events. Tests revealed that none of the patients had a deficiency in natural anticoagulants AT, protein C and protein S, nor did they have elevated Factor VIII levels; no lupus anticoagulant was detected. DNA from blood samples were analyzed [6].

One of the investigated elements in this study was MTHFR polymorphism. Hyperhomocysteinemia is caused by the decreased activity of MTHFR and may increase the risk for arterial and venous thrombosis in individuals with folic acid deficiency. Decreased MTHFR activity is a consequence of two common C677T, A1298C substitutions which makes the enzyme thermolabile. Evidence for an association of RVO with hyperhomocysteinemia has been highlighted in isolated studies and meta-analysis. Even though the Giannaki et al. study shows that the prevalence of MTHFR polymorphisms does not appear to be significantly different between patients with RVO and controls, it is important to notice that 80.3% of patients carried at least one affected MTHFR allele, so that MTHFR involvement in RVO pathogeny cannot be ruled out [6].

The MTHFR gene may undergo different mutations (or variants). The most frequent one, C677T, is linked to hyperhomocysteinemia and occlusive vascular pathology. Mutation A 1298C is still not very well studied. What is known is that the A1298C polymorphism of the MTHFR gene refers to:

− substitution of adenine (A) with cytosine (C) in the position 1298 in the MTHFR gene area on the DNA chain.
− Substitution of glutamic acid with alanine in the position 429 in the polypeptide chain of the enzyme (Glu429Ala).

Polymorphism in the MTHFR gene may decrease the activity of methylenetetrahydrofolate reductase, leading to a mild increase of homocysteine in the blood [7]. This may cause problems within blood vessels and is considered a risk factor for thromboembolism. However, many studies show that there are individuals with MTHFR mutations who have normal homocysteine levels, and there are not at risk for developing blood clots. Thus, the MTHFR mutation by itself does not cause clotting disorders, but the coexistence of different mutations may furthermore increase the risk for thromboembolic accidents [8].

Symptoms caused by MTHFR mutations may vary among individuals and depend on the type of mutation. Patient usually are not aware they have an MTHFR mutation unless they experience severe symptoms or undergo genetic testing. One or two MTHFR mutations can slightly increase the levels of homocysteine in the blood. Depending on how severe this condition is, the possible consequences may be abnormal blood clotting, seizures, poor coordination, anemia, cardiovascular diseases, strokes, heart attacks, depression, or behavior disorders [9,10].

Vieira et al. studied MTHFR polymorphism, aiming to highlight thrombotic and cardiovascular risk factors (CRF) for retinal vein occlusions [11]. The retrospective study analyzed 60 consecutive case series of patients with RVO, tested for CRF, hyperhomocysteinemia (HH), lupus anticoagulant, antiphospholipid antibody and 5 gene variants: factor V Leiden (G1691A), factor II (PT G20210A), 5,1-methylenetetra-hydrofolate reductase (MTHFR; 677 C > T and 1298 A > C), plasminogen activator inhibitor 1 (PAI-1; 4 G/5 G). More than 1 CRF was present in 60% of the patients, which had a significantly higher mean age at diagnosis (66.7 ± 12.9 versus 59.5 ± 13.7 with ≤1 CRF, [t(57) = −2.05, *p* = 0.045, d = 0.54]). Patients with thermolabile MTHFR forms with decreased enzyme activity (T677T or C677T/A1298C) had a significant lower mean age (57.6 ± 15.1) than patients with normal MTHFR enzyme activity (68.5 ± 10.2). The study also highlights the fact that, although severe HH is rare, mild HH occurs in approximately 5% of the general population (mild HH is an independent risk factor for venous thromboembolism) and that there may be an association between HH, coronary heart disease, deep venous thrombosis and CRVO. MTHFR compound heterozygosity C677T/A1298C was found in 26.7% of patients. MTHFR polymorphisms were found in 55 patients (91.7%) [11].

In recent years, studies have failed to prove a link between RVO and Factor V Leiden, being in contradiction to a meta-analysis published by Rehak et al. (2010) which indicated a possible relationship between this common mutation and retinal vein thrombosis [11].

Factor V Leiden is present in the 5% of the healthy population, but its presence may vary on regional grounds. For example, this factor is more frequent in northern Europe than in southern Europe, but it is not present in the Chinese and Japanese population, as well as in the native Australian and African. Regarding other mutations, such as prothrombin G20210A, its presence in healthy European Caucasian population is about 1% to 8%; in patients with confirmed venous thromboembolism episode (VTE) it is 3% to 17%. A study published in 2011 showed that the angiotensin-converting enzyme (ACE) gene variant was higher in native Americans than in Caucasians or Africans [12].

Special attention should be paid to the PAI-1 (Plasminogen activator inhibitor type 1) 4G allele mutation. Several meta-analyses pointed out an association between PAI 1 4G and both rheumatoid arthritis and cardiovascular risk [12].

Present data show that plasminogen activator inhibitor 1 (PAI-1) polymorphism was similar among different people, while the distribution of C677T allele showed geographical and ethnic variations. In African Americans, Asians, and Africans there this mutation has a low frequency. The 4G/4G polymorphism of PAI-1, associated with high plasma level of PAI-1 activity, is a risk factor for coronary heart diseases and venous thrombosis, especially in patients with other genetic thrombophilia mutations. The prevalence of hereditary thrombophilia is known in patients with lower limb thrombosis or pulmonary embolism but not clearly confirmed in patients with thrombosis in other sites. In a study published by Bombeli et al., 39% of the patients with lower extremity thrombosis presented thrombophilia, but these genetic mutations were found only in the 5.9% of patients with RVO [13].

From the existing data so far, it appears that the role of thrombophilia in patients with RVO may be more important in patients without acquired risk factors such as diabetes, hyperlipidemia or hypertension. Another aspect to consider is that thrombophilia might play more important role in younger people with RVO than in older people. Overall, the role of thrombophilia in patients with RVO seems to be still very controversial [14].

Even though the plasminogen activator inhibitor-1 (PAI-1) 4G/5G polymorphism has been associated with increased risk of venous thromboembolism, the existing evidence is still scarce. Different meta-analyses have been performed, to verify and possibly prove this connection.

Wang et al. developed a systematical search in PubMed, EMBASE, Wanfang, China National Knowledge Infrastructure (CNKI) and CQVIP databases to identify relevant studies published before March 6th, 2014. The odds ratios (ORs) with 95% confidence intervals (CIs) were pooled using the fixed/random-effects model using Review Manager 5.1 and STATA 12.0. A total of 34 studies with 3561 cases and 5693 controls were analyzed. The studies included in this research contained both Caucasian and Asian patients. Summing up, a significant association between the PAI-1 4G/5G variant and VTE risk in total population has been observed. This variant was also related to the deep vein thrombosis risk. In the subgroup analyses on ethnicity, significant results were obtained in both Asians and Caucasians. Regarding subgroup analyses about coexistence of other thrombotic risk factors, the PAI-1 4G/5G polymorphism was significantly associated with venous thrombosis risk in patients with Factor V Leiden mutation, but not in patients with cancer or surgery. The results of this meta-analysis point out the role of PAI-1 4G/5G polymorphism as a risk for VTE susceptibility, especially in patients with other genetic thrombotic disorders [15].

Another side of these genetic disorders to be taken into consideration is that PAI-1 is the main inhibitor of fibrinolysis; according to the latest studies, high levels of PAI-1 may increase the risk of cardiovascular disease. The 4G/5G polymorphism affects PAI-1 gene transcription with lower levels of plasma PAI-1 in the presence of the 5G allele [16].

Regarding the role of the fibrinolytic system in the pathogenesis of RVO, it appears that 4G/5G gene polymorphism, due to an insertion/deletion (I/D) of a G in the promoter of the PAI-1 gene, is associated with thromboembolic events. Homozygosity for the 4G allele is associated with 25% higher levels of PAI-1 and may have an additive effect to the risk for venous thromboembolism (VTE), when there are other environmental or genetic risk factors [17]. Concerning the ocular onset of thrombosis, it is believed that increased levels of PAI-1 might be a risk factor for RVO in a subgroup of patients without established factors for RVO or factors that influence PAI1 activity. The study of Turello et al. revealed that there is a high prevalence of the 4G allele (84.7%) in patients with RVO compared to control group, although it did not correlate with PAI-1 levels, while Glueck et al. found an association with the 4G allele and RVO and a higher PAI-1 activity in cases than controls [17,18,19].

The Giannaki et al. analysis regarding implications of genetic predisposition in RVO pathogeny implies that it is uncertain to what degree hypercoagulability is involved in the development or recurrence of RVO, since patients with history of thrombosis may be more at risk, especially when having acquired risk factors and an old age. It has been suggested that factors that predispose to arterial rather than venous thrombosis are implicated in RVO pathogenesis, therefore further studies are necessary on this matter [6].

Since coexistence of thromboembolic genetic factors and several comorbidities are involved in RVO pathogenesis, we need to pay a special attention on ocular risk factors that may be associated to RVO, such as ocular hypertension and, particularly, chronic glaucoma [1,20,21].

## 3. Considerations on Genetical Factors Linking Glaucoma Association with RVO

Glaucoma is a multifactorial optic neuropathy with environmental and genetic causes and a leading cause of blindness worldwide. In the glaucomatous disease, there is a characteristic loss of nerve fibers, which leads to progressive and eventually irreversible loss of vision. Primary glaucoma is classified in primary open angle glaucoma (POAG), primary congenital glaucoma (PCG) and primary angle-closure glaucoma (PACG). There are several studies that support that genetic polymorphism, such as the variant MTHFR A1298C, may increase the risk for developing glaucoma, especially in the heterozygote model [9,22]. However, while there are meta-analyses supporting this idea, other studies are yet to be extended, given the fact that, while some of these studies tried to maintain a heterogeneity of the assessed patients, other studies were made on specific ethnic groups [7].

In addition, latest studies also report that there may be a strong connection between primary angle closure (PAC) or primary angle closure glaucoma (PACG) and RVO (mainly CRVO). This association could be explained not only by genetic predisposition, but also by the mechanical changes in the lamina cribrosa in the eyes with PAC/PACG that contribute to the onset of RVO. PACG combined with the genetic polymorphism of such patients may additionally increase the risk for RVO/CRVO onset [23].

As previously pointed out, RVO has been associated with many general conditions, such as older age, ischemic heart disease, diabetes, or high blood pressure; yet the genetic connection between glaucoma and RVO is still a controversial topic. The relationship between RVO and neovascular glaucoma (NVG) has already been established. NVG may develop either after BRVO, HRVO or CRVO. Regarding other types of glaucoma, it appears that the onset of BRVO and CRVO is more frequent in patients with glaucoma. Since the 90’s, POAG has been especially linked to RVO. However, there is still a need to properly investigate the connection between different types of RVO and different types of glaucoma, considering all data available are either insufficient or misleading [7].

Yin et al. (2019) carried out a meta-analysis summarizing epidemiological evidence on the association between glaucoma and the risk of retinal vein occlusion (RVO). Relevant studies were selected by searching in PubMed, EMBASE and Cochrane until February 2018. The analysis included 15 eligible observational studies. All results were carefully examined and pooled using random effects models with 95% confidence intervals (CI) [24].

In the random effect model, glaucoma was found to be a risk factor for RVO in all 15 studies with high methodological quality. This situation may be explained by different hypotheses. Kim et al. (2011) suggested that the pathogenesis of RVO is most probably associated with glaucomatous anatomic changes [25]. Moreover, even before that, Sonnsjo & Krakau (1993), revealed a vascular hypothesis of glaucoma [26], confirmed by Gao et al. (2015) [27]. Glaucoma subjects, including those with primary angle closure glaucoma and primary open angle glaucoma, have narrower retinal arteries and veins than normal individuals. Therefore, RVO may occur because of glaucomatous structural changes or may coexist with retinal hemodynamic anomaly.

In advanced subgroup analyses, glaucoma was proven to be associated with RVO to varying degrees, depending on its type (CRVO, BRVO and HRVO). It has been postulated that elevated intraocular pressure may compress vessel walls and cause blood vein intimal proliferation, which leads to the collapse of retinal capillaries. Moreover, open angle glaucoma precedes vascular occlusion [28,29,30,31]).

Patients with glaucoma may often present disc hemorrhage. Since disc hemorrhages are, in fact, small vein occlusions, it has been hypothesized that glaucoma, especially OAG, retinal vein occlusion and disc hemorrhage might have a common pathogenesis [32]. One fact to consider is that retinal vessel parameters are very different for PACG and POAG. It has been shown that vessel caliber and retinal vessel oxygenation in PACG are relatively higher than POAG and NTG [33]. Another fact is that the pathogenesis of PACG and POAG is different. In PACG, the shallowness of the anterior chamber and the narrow irido-corneal angle may be the main trigger for RVO (Jonas et al.2013, Mohammadi et al., 2015) [34,35]. There are not many studies on RVO in PACG, so significant associations between PACG and RVO risk are yet to be found. More large-scale prospective studies with PACG and RVO incidence are still warranted to clarify the association. Of all 15 studies, 6 differentiated the RVO subtype (CRVO, HRVO and BRVO), and 5 classified the glaucoma subtype (PACG and POAG/COAG). In these studies, glaucoma was linked to the development of CRVO, while this association appears to be less clear for BRVO. There were only two studies that found a relationship between RVO subtypes and glaucoma subtypes. In these two studies, OAG (POAG/COAG) was found to be a significant risk factor for CRVO, whereas PACG was related to CRVO (OR: 5.3; 95% CI:1.04–26.95; *p* = 0.045) but not significantly to BRVO (OR: 0.65; 95% CI: 0.07–6.27; *p* = 0.707). Hayreh (2004) and Hayreh et al. (2005) supposed that primary angle closure may cause RVO through a mechanism similar to that of POAG [32,36]. In a retrospective study, angle closure was hypothesized to be associated with retinal vein occlusions, especially CRVO/HRVO (Michaelides & Foster 2010) [37]. Some studies show that there is a higher frequency of PAC/PACG in RVO than in the general population, while the frequency of PAC/PACG in BRVO (3.1%) was similar to that of the general population (3.9%) [38,39]. The same meta-analysis concludes that there may be a plausible relationship between PACG and BRVO risk, but PACG is more closely related to CRVO risk than to BRVO risk.

To sum up the results of the meta-analysis, it was demonstrated that glaucoma is a significant risk factor for RVO; it appears that OAG (POAG/COAG) may increase the incidence of RVO, especially of CRVO, while there was less association between PACG and RVO, especially for BRVO. However, there are yet many studies necessary to draw a conclusion on this matter [24]. Therefore, the implications of various hematological abnormalities in the etiopathogenesis and treatment of CRVO remain partially unclear and there are contrasting clinical results [3].

## 4. A Possible Pathological Relation That Raises a Question: Is There a Genetic Link between Rheumatoid Arthritis (RA) and Cardiovascular (CV) Factors?

While assessing thromboembolism, one should consider the general medical background of the patient, for example, the existence of an inflammatory autoimmune disorder, such as rheumatoid arthritis; in this case, the risk of developing CRVO may increase. Studies show that there is an increased risk of developing venous thromboembolism for RA patients compared with non-rheumatoid arthritis patients. The same studies show that the risk can be attenuated, but it remains elevated even after adjusting for various risk factors for venous thromboembolism [40].

RA is a condition that places patients at risk for developing cardiovascular events due to accelerated atherosclerosis. RA is, as well, an entity that causes chronic inflammation; this fact, along with the genetic background, may increase the risk of cardiovascular events, regardless of the presence of traditional cardiovascular risk factors. It is a topic that needs to be explored, considering that patients with RA are particularly exposed to cardiovascular risk; cardiovascular diseases in such patients appears to be the most common cause of premature mortality. The main cause involved in this outcome may be the result of an accelerated atherosclerotic process. Both RA and atherosclerosis are complex polygenic diseases, and both are chronic inflammatory diseases with similar pathophysiological mechanisms [22], which display a strong genetic component of susceptibility. RA has an estimated heritability of up to 60% and cardiovascular disease in the general population of up to 30%–60%. In addition, a specific genetic background may contribute to the development of both diseases [12,15,41].

Several studies have already confirmed the role of genetic factors in the development of atherogenesis in RA patients. Nevertheless, despite research effort to unlock the genetic basis of cardiovascular disease in RA, further studies are still necessary to further establish the genetic influence in the increased risk of cardiovascular events observed in patients with RA [41].

As mentioned before, hyperhomocysteinemia is considered to be an independent nontraditional risk factor for cardiovascular disease, especially coronary disease, in the general population. It is well known that homocysteine is an intermediary amino acid resulting from the conversion of methionine to cysteine. Increased levels of homocysteine may occur in some uncommon autosomal defects of the metabolizing enzymes 5,10-methylene tetrahydrofolate reductase (MTHFR) and cystathionine β-synthase. Less severe elevations of homocysteine levels are more frequently seen as an outcome of either heterozygous mutations of these enzymes or dietary deficits of vitamin B12 or folate or in patients with impaired renal or liver function. Homocysteine has a direct toxic effect on endothelial cells, having, among other effects, a prothrombotic impact.

Most of the patients with RA are known to have high levels of homocysteine. Among the medication that these patients receive, Methotrexate (MTX) in particular is found to lower cardiovascular risk. This treatment is a two-edged sword, since there are some effects of MTX in decreasing red blood cell folate, thus resulting in increased levels of homocysteine, due to MTHFR reduced activity. In these patients, folic acid supplements may have a beneficial effect [42].

Research data show that there is a common C677T polymorphism in the gene coding for the MTHFR enzyme that may contribute to the genetic risk factor for cardiovascular disease in the general population. However, there is also the A1298C polymorphism in the MTHFR gene that has been associated with MTHFR activity. The A1298C polymorphism apparently has a lower effect in reducing enzyme activity, compared with the 677 mutation. This aspect is better observed in the homozygous (CC) condition than in the heterozygous (AC) or normal (AA) states. Heterozygote subjects for the C677T and the A1298C mutations are known to display 50% to 60% of control activity, a value significantly lower than that in the case of single heterozygotes for the C677T variant. Recently, it has been revealed that there is an association of the A1298C polymorphism in the MTHFR gene with susceptibility to RA in Southern European individuals.

Considering all the above, many studies aim to prove whether there is a potential contribution of the MTHFR 677 C>T and 1298 A>C gene polymorphisms to disease susceptibility of patients with RA or if it may be correlated with an increased risk of subclinical atherosclerosis manifested by the presence of endothelial dysfunction in RA [41,42].

Since the role of homocysteine in the mechanism correlated with increased risk of cardiovascular events in the general population is acknowledged, functional polymorphisms in the MTHFR gene have been analyzed, as potential candidates for atherosclerosis in RA, which is associated with increased risk of cardiovascular events and mortality. Palomino-Morales et al. were able to link for first time an involvement of the MTHFR A1298C gene polymorphism in the higher risk of atherosclerosis of patients with RA. The study was able to prove that there is a connection between the mutant allele C of the MTHFR A1298C polymorphism with increased risk of cardiovascular events. Furthermore, subjects carrying the MTHFR 1298AC and MTHFR 1298CC genotypes were found to have a more severe endothelial dysfunction; this finding was also linked to subclinical atherosclerosis in patients with RA. Other studies showed that the MTHFR allele 1298C has been found to be associated with a risk of early-onset coronary artery disease independent of homocysteine, folic acid, or vitamin B12 levels [43]. On this matter, Weisberg and colleagues reported that the MTHFR 1298 mutation alone does not affect plasma homocysteine levels [44].

The molecular pathology of the missense A1298C mutation remains unknown. This mutation consists in an amino acid change of glutamate to alanine in the regulatory C-terminal domain of the enzyme and is not associated with classic cardiovascular risk factors or manifested by hyperhomocysteinemia. Even though the molecular mechanism of this mutation remains unknown, we do know that in vitro, MTHFR 1298C carriers exhibit decreased enzyme activity, which indicates the functional importance of the A1298C polymorphism [44].

The study of Palomino-Morales et al. shows that it seems that this polymorphism is only associated with an increased risk of cardiovascular disorders under low-folate conditions, varying between different populations according to characteristic folate intake. This study showed for the first time an association of MTHFR A1298C gene polymorphism with the risk of cardiovascular events and subclinical atherosclerosis manifested by the presence of endothelial dysfunction in patients with RA, but other studies are still necessary to clarify this issue [42].

Detecting RVO and associated systemic and local conditions as early as possible may strongly improve the visual outcome of the patient and both the long-term visual acuity and ocular morbidity. It is crucial, when encountering a patient with such a complex pathology, to explore all the possible causes, to treat them promptly and efficiently, to be able to prevent further vascular occlusions and to preserve a good long term visual acuity.

## 5. Discussions

We present the case of a 56-year-old woman, previously known, with several miscarriages (25 years prior), rheumatoid polyarthritis (RA) diagnosed at 38, poorly controlled systemic blood pressure. The patient presented to the ER with severe headache and blurred vision in the right eye, with sudden onset, 2 days prior.

Upon admission, the best corrected visual acuity in both eyes was 20/20 (0 logMAR), with difficulty.

Intraocular pressure was 65 mmHg in the right eye and 31 mmHg in the left eye (by Goldmann aplanotonometry).

Slit lamp examination of the anterior segment highlighted epithelial corneal edema in the right eye, anterior chamber depth significantly decreased in both eyes, and a non-reactive semi-mydriatic pupil in the right eye, while in the left eye the pupil was miotic and reactive.

**Figure 1:**
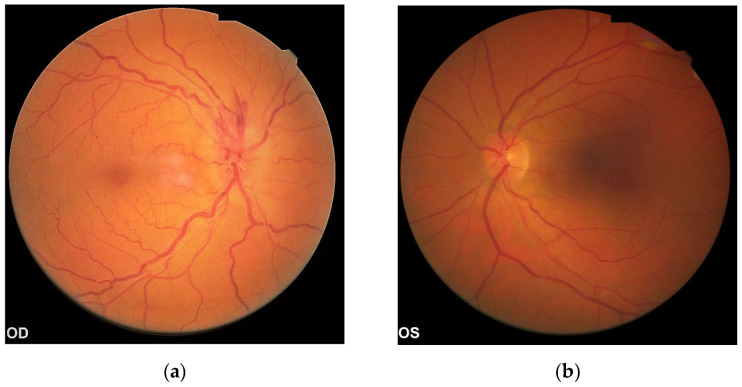
Fundus Examination of the right eye: (**a**) Right Eye; (**b**) Left Eye.

Fundus examination revealed an elevated hyperemic blurred optic disc in the right eye, with a suprajacent flame shaped hemorrhage, tortuous and engorged retinal veins, macula with foveolar reflex (Figure 1a). C/D ratio could not be estimated; in the left eye, the optic disc had a normal aspect, with cup/disc ratio 0.2, a normal aspect macula and normal configured retinal vessels (Figure 1b).

**Figure 2:**
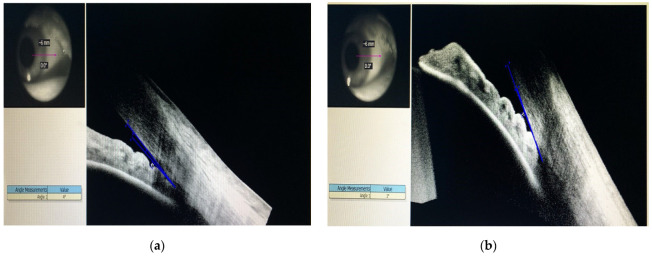
Gonioscopy in both eyes, supported by the anterior segment OCT: (**a**) Right Eye; (**b**) Left Eye.

Gonioscopy showed angle closure in both eyes, supported by the anterior segment OCT that revealed a 4 degree closed angle in the right eye and a 1 degree closed angle in the left eye (Figure 2a,b).

Based on the clinical and paraclinical aspects, the preliminary diagnosis was central retinal vein occlusion (in the right eye) and bilateral acute angle closure glaucoma.

The patient was administered osmotic diuretic iv (Mannitol 15%) 250 mL, oral carbonic anhydrase inhibitors, antiplatelet drugs (Aspirin), Sulodexide 250 USL, Clexane 2000 UI/day injected subcutaneous, and received eye drops with beta-blockers and carbonic anhydrase inhibitors. After lowering the intraocular pressure, pilocarpine 2% eye drops were instilled and laser iridotomy was performed at 12 o’clock on each eye, followed by the stabilization of the IOP within normal values.

Given the fact that our patient had an autoimmune inflammatory disease, a history of miscarriages, which added to the ocular pathology, further examinations were performed. Cardiovascular exam highlighted, at the time of the examination, elevated blood pressure (for which a conversion enzyme inhibitor has been recommended), and cord ultrasonography was within normal limits. The blood exam revealed elevated levels of CRP 158.4% (normal range 70%–140%) and slightly elevated levels of free Protein S, 116.1% (normal range 60.1%–113.6%). The resistance to activated C protein was 1.14% (normal range >0.8%). The blood test was performed by Protein C global assay and indicated Factor V Leyden—normal; thus, it excluded any type of anomaly that increased resistance to activated C protein, including any anomaly of Factor V Leyden. Coagulation tests (APTT, PT, Fibrinogen, ESR) were normal. CBC (except a small increase in hemoglobin (14.8 g/dL)) and the biochemical profile were within normal range, and a series of additional tests were performed, to exclude a possible infectious cause, which were all negative. Antibodies associated with acquired thrombophilia (Anti-phospholipidic antibodies, Anti-cardiolipin antibodies, Beta 2 glicoprotein-1 antibodies, lupus anticoagulant) were negative as well.

Based on the clinical and paraclinical examinations, the final diagnosis was upper hemi retinal vein occlusion (in the right eye), bilateral acute closed angle closure glaucoma, rheumatoid polyarthritis, systemic arterial hypertension.

The differential diagnosis was a challenge (Table 1). We suspected the occlusive cause for many reasons. First, we excluded anterior ischemic optic neuropathy; eye vision was 20/20, no visual field loss, no history of diabetes mellitus or dyslipidemia; the inflammatory markers were low, excluding a possible arteritic cause. Papillophlebitis was also not an option, considering that it usually appears at a younger age and there were no infectious, inflammatory, or demyelinating findings. Diabetes mellitus was also excluded, given the normal blood sugar levels, and the clinical fundus aspect, which was normal in the left eye. Ocular ischemic syndrome was also removed, given the clinical aspect and the normal carotid Echo Doppler exam along with the exclusion of dyslipidemia and vascular stenosis [45,46]. The reasons that finally tipped the balance in favor of the upper hemi-retinal vein occlusion (instead of CRVO) were the arcuate inferior defect in the visual field, along with the upper flame hemorrhages.

Taking in consideration the medical history of the patient, especially the rheumatoid arthritis, the miscarriages and the CRVO, genetic tests were performed, to further investigate the patient complex condition.

DNA was extracted from the peripheral blood sample. Twelve gene mutations and alleles that have been associated with the genetically determined risk of thrombophilia and/or cardiovascular diseases were screened using multiplex PCR with biotinylated primers followed by a reverse hybridization assay (CVD Strip Assay, IVD). The mutations screened using this technique were: factor V Leiden (1691G>A; R506Q), Factor V H1299R (R2), Prothrombin (PTH; Factor II) 20210G>A, 5,10-Methylenetetrahydrofolate reductase (MTHFR) C677T and A1298C, Factor XIII V34L, Plasminogen Activator Inhibitor 1 (PAI-1, Serpin E1) 4G/5G, Beta-Fibrinogen (FGB)- 455G>A, Angiotensin-Converting Enzyme (ACE) 287 bp insertion/deletion (I/D), Human Platelet Antigen 1 (HPA 1 a/b; GpIIIa, integrin beta 3 L33P), Apolipoprotein B (Apo B) R3500Q, Apolipoprotein E (Apo E) E2/E3/E4. The results of the genetic testing of the patient detected PAI-1 4G allele and MTHFR A1298 C mutation in heterozygosity, indicating an increased risk for thromboembolic incidents.

The patient was followed-up monthly and remained on lowering IOP medication—a combination of prostaglandin analogue and beta-blocker was administered once a day, permanently. The patient maintained a good visual acuity and a good clinical aspect of the eye fundus, as well as a good optic nerve and macular OCT aspect; the visual field reveals a small inferior arcuate defect.

One of the first important issues to consider in this case was to decide if in our patient case, there was a central retinal vein occlusion, which was revealed in its early stages, or an upper hemi-retinal vein occlusion. Even though all venous branches were engorged and dilated, and the optic nerve was swollen, the fact that there were visual field changes inferiorly, correlated with hemorrhages in the upper quadrants, led us to believe there was an upper hemi retinal vein occlusion [45,46]; most likely, it was preceded by the angle closure, and the lowering of the IOP, together with anticoagulant and antiaggregant treatment prevented further development of this condition.

The features of this case are the sudden onset of bilateral acute angle closure, along with the hemi retinal vein occlusion in a hypertensive patient with rheumatoid polyarthritis and with a history of multiple miscarriages, whose genetic tests revealed specific mutations (MTHFR A1298C and PAI-1 4G heterozygote) that increase not only the risk for developing venous thromboembolism, but also the risk of developing chronic glaucoma.

In our patient case, the mutation MTHFR A1298C indicates a heterozygote mutation, which means that there is a mutation on just one chromosome out of two. This kind of mutation may cause a decrease in the enzymatic capacity of up to 20%, with slightly elevated levels of homocysteine. That, combined with PAI-1G mutation, may submit the patient to an even higher risk of blood clotting, even further in the future.

Another interesting aspect of this case is the possible association between rheumatoid polyarthritis and thrombosis. Studies reveal that the MTHFR 1298 A>C gene polymorphism may increase the risk for subclinical atherosclerosis and cardiovascular episodes in patients with RA. As already pointed out, patients with rheumatoid arthritis are more exposed to the cardiovascular risk due to the accelerated atherosclerosis they may develop. Adding genetic disorders to the chronical inflammation increases again the risk of thromboembolic events, regardless of the presence of usual cardiovascular risk factors [42,47].

There are also issues to consider regarding the possible connection between primary angle closure (PAC) or primary angle closure glaucoma (PACG) and retinal vein occlusion. As previously shown, this association may be explained not only by genetic predisposition, but also by mechanical changes on lamina cribrosa in eyes with PAC/PACG; these changes are believed to contribute even further to the onset of RVO. Studies also show that PACG combined with the genetic polymorphism of such patients may increase even further the risk for RVO/CRVO onset, which may explain the clinical condition of our patient [23].

## 6. Conclusions

There is an increased number of studies regarding genetic polymorphism and its implications in different pathologies, such as cardiovascular and/or autoimmune conditions. Coexistence of these conditions with several genetic factors may increase one’s risk of developing thromboembolic events in the ocular area, especially in patients with associated ocular diseases such as glaucoma.

There are studies implying that genetic polymorphism, such as variant MTHFR A1298C, may increase the risk for developing glaucoma, especially in the heterozygote model [9,22]. However, in order to support this idea, extended studies are still necessary [7].

Other studies raise the question of the association of the A1298C polymorphism in the MTHFR gene with the susceptibility to RA in Southern European individuals, and if there is a connection between genetic polymorphism, RA and the increased risk of subclinical atherosclerosis manifested by the presence of endothelial dysfunction in RA [41,42]. Subclinical atherosclerosis may be a trigger for thromboembolic events such as RVO.

Complex clinical cases such as the one presented in this paper increase the awareness regarding not only thromboembolic ocular events, but also their association with other ocular pathologies, and with general disorders. These cases require a wide approach, both clinically and thorough research in order to establish the best treatment and follow up for the patient, to preserve as much as possible a good visual outcome and a satisfactory health status, and to approach patients in all the complexity of their pathologies.

## Figures and Tables

**Table 1:** The differential diagnosis of the case.

Central/Retinal Vein Occlusion (CRVO)	Papillophlebitis	Ocular Ischemic Syndrome	Papillitis	Anterior Optic Ischemic Neuropathy	Diabetic Retinopathy	Hypertensive Retinopathy
Age: variable Usually, history of vascular disease	Age: <50 (Usually women 25–35 y.o.)	Age: 50–80	Age: Variable	Non-arteritic Age: >50 Arteritic Age: >50	Age: Variable, usually 50–80	Age: Variable
Unilateral	Unilateral	Uni/bilateral	Unilateral	Unilateral	Bilateral	Bilateral
Optic disc: edema	Optic disc: edema	Optic disc: normal	Optic disc: hyperemic edema	Non-arteritic Optic disc: hyperemic edema Arteritic Optic disc: edema, usually with pallor.	Optic disc: normal/rarely diabetic papillopathy	Optic disc: normal/edema
**Other features**	**Other features**	**Other features**	**Other features**	**Other features**	**Other features**	**Other features**
Macular edema Veins: dilated and tortuous Hemorrhages: flame shaped, in all quadrants/in nerve fiber layer/ in the affected quadrant, +/− cotton wool spots/rarely hard exsudates	Clinical features of CRVO, no history of vascular disease Misdiagnosed, may lead to CRVO with macular edema and poor acuity.	Veins- dilated Hemorrhages: dot, in mid periphery and deep retinal layers Microaneurysms	Usually related to inflammatory/ infectious/ demyelinating diseases	Non-arteritic History of vascular disease/diabetes/dyslipidemia Central scotoma/altitudinal defects Arteritic: flame shaped hemorrhage adjacent to the optic disc. Increased inflammatory markers (ESR, CRP, fibrinogen) Jaw claudication	Veins: dilated, tortuous, beaded, Hemorrhages: dot/blot, posterior pole/mid periphery Microaneurysms Intraretinal microvascular anomalies, all over the retina	Macular edema in advanced stages Veins: dilated. tortuous Salus/Gunn/Bonet signs Hemorrhages, retinal hard exudates, or cotton wool spots

## Data Availability

No new data were created or analyzed in this study. Data sharing is not applicable to this article.
